# TECHNICAL ASPECTS OF LAPAROSCOPIC SLEEVE GASTRECTOMY

**DOI:** 10.1590/S0102-6720201500S100018

**Published:** 2015-12

**Authors:** Almino Cardoso RAMOS, Eduardo Lemos de Souza BASTOS, Manoela Galvão RAMOS, Nestor Tadashi Suguitani BERTIN, Thales Delmondes GALVÃO, Raphael Torres Figueiredo de LUCENA, Josemberg Marins CAMPOS

**Affiliations:** 1 Gastro-Obese-Center - Advanced Center of Gastroenterology, Metabolic and Bariatric Surgery, São Paulo, SP; 2 Federal University of Pernambuco, Recife, PE), Brazil

**Keywords:** Morbid obesity, Gastrectomy, Laparoscopy

## Abstract

***Background*:**

The vertical gastrectomy indications for surgical treatment of morbid obesity have increased worldwide. Despite this increase, many aspects of surgical technique still remains in controversy.

***Aim*:**

To contribute presenting surgical details in order to better realize the vertical gastrectomy technique in bariatric surgery.

***Methods*:**

Technical systematization, patient preparation, positioning of the trocars, operative technique and postoperative care are presented in details.

***Results*:**

During 12 months were enrolled 120 patients undergoing GV according to the technique described herein. The results are published in another ABCD article (ABCD 2015;28(Supl.1):61-64) in this same volume and number.

***Conclusion*:**

The surgical technique proposed here presented itself viable and facilitating the surgeon's work on difficult points of the vertical gastrectomy.

## Abstract (pt)

**Racional:**

: As indicações da gastrectomia vertical no tratamento cirúrgico da obesidade mórbida têm aumentado no mundo todo. Apesar deste aumento, diversos aspectos relativos à técnica operatória têm sido controversos.

**Objetivo:**

: Contribuir com detalhes cirúrgicos afim de melhor a técnica da gastrectomia vertical na cirurgia bariátrica.

**Métodos:**

: A sistematização técnica, o preparo do paciente, o posicionamento dos trocárteres, a técnica operatória e cuidados do pós-operatório são apresentados em detalhes.

**Resultados:**

: Durante 12 meses foram incluídos 120 pacientes submetidos à GV de acordo com a técnica aqui descrita. Os resultados estão publicados em outro artigo do ABCD (ABCD 2015;28(Supl.1):61-64) neste mesmo volume e número.

**Conclusão:**

: A técnica cirúrgica aqui proposta apresentou-se viável facilitando o trabalho do cirurgião em pontos difíceis da gastrectomia vertical.

## INTRODUCTION

The apparent technical simplicity of the sleeve gastrectomy (SG) is due to the fact that the technical steps are based on only in one organ, approach in supramesocolic abdominal quadrant, and without conducting endosutures or anastomoses between different organs. However, there are several technical details that are not yet in consensus among bariatric surgeons, even the most experienced. The calibration of the remaining gastric tube, conducting oversuture reinforcing the staple line, distance from the pylorus to start clipping, and resulting gastric amount in antrectomy, are in constant discussions. Besides the technical aspects, there is also no consensus on the indications, the metabolic effects and the long-term results. Regarding complications, fistula in the line of staples although with incidence rate similar to Roux-en-Y gastric bypass, generally carries higher morbidity and healing time; based on this, it is of great concern among surgeons^2^^,^ 3 ^,^ 4 ^,^ 10.

Thus, despite the SG already is a common bariatric procedure also entails studies needing to clarify controversial aspects regarding the surgical technique. Therefore, this study aims to contribute with technical details that can improve and make easier the use of SG in bariatric surgery.

## METHOD

### Technical systematization 

### Preparation

The operation is performed with the patient supine, with open legs in reverse Trendelenburg position (inclined) in operating table with angle of 30°. The main surgeon is positioned between the lower limbs; the assistant surgeon and the scrub nurse are on his right side. To prevent fall injuries and poor positioning, patients are attached to the operating table with the use of special braces in the abdomen and lower limbs. Pneumoperitoneum is performed with direct abdominal puncture with a Veress needle in the left upper quadrant, along the costal margin in the midclavicular line, kept up with inflation pressure of 16 mmHg and flow 40 l/min of carbon dioxide. Before the operation is applied by subcutaneous enoxaparin 40 IU, antibiotic prophylaxis with 2 g of cefazolin and introduced gavage-caliber (32 Fr) orally, positioned a few centimeters below the gastroesophageal transition.

### Positioning of the trocars

**FIGURE 1:**
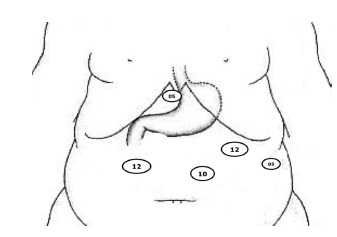
- Positioning of the trocars for laparoscopic SG

Taking as reference the average anatomical xifoumbilical line, the first trocar (10 mm, permanent) is inserted at the intersection of two thirds with the lower upper third of about 3 cm to the left of the patient. This position allows frontal approach to gastroesophageal transition without the risk of puncture stand in the middle of the round ligament (trocar 1 - T1). The second trocar (5 mm, permanent) is placed next to the xiphoid process (trocar 2 - T2) for removal of the liver. The third (12 mm, disposable) is positioned on the right side of the patient, at the right midclavicular line in parallel to the T1 (trocar 3 - T3). The fourth (5 mm, permanent) is placed in the left anterior axillary line along the costal margin (trocar 4 - T4). The last trocar (12 mm, disposable) is placed at the level of the left midclavicular line, also near the costal margin (trocar 5 - T5) (Figure 1).

### Surgical technique

**FIGURE 2:**
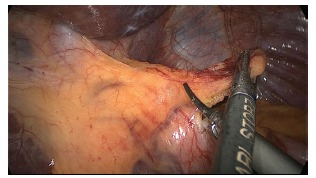
- Removal of the fat pad near the esophagogastric junction

**FIGURE 3:**
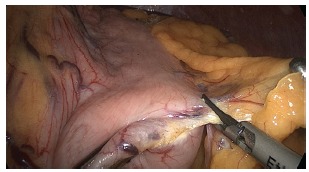
- Dissection of the great gastric curvature in the proximal direction to the oesophagogastric angle

**FIGURE 4:**
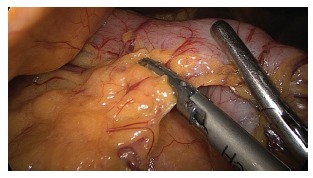
- Dissection of the great gastric curvature distally up to 2 cm from the pylorus

**FIGURE 5:**
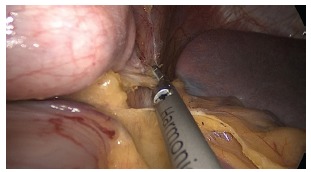
- Full release of the gastric fundus near the diaphragmatic crus

**FIGURE 6:**
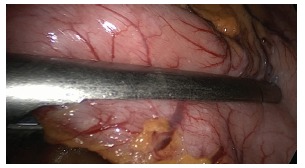
- Mechanical stapler positioned in the antrum next to the pylorus for the first shot

**FIGURE 7:**
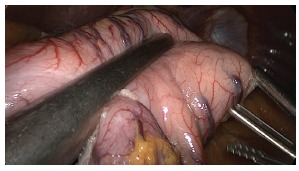
- Mechanical stapler positioned for early gastric body tubing with gastric tube 32 Fr modeling the stomach

**FIGURE 8:**
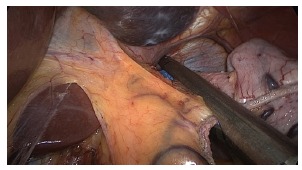
- Mechanical stapler positioned near the gastroesophageal transition to perform the last shot

The operation begins with the dissection and removal of the fat pad of the esophagogastric junction (Figure 2), to allow complete visualization of the left face of the left diaphragmatic crus. Then proceeds to release and ligation of the great gastric curvature with ultrasonic energy (Ultracision Harmonic Ace Plus - Ethicon - Johnson & Johnson Corporation - USA) starting at the distal portion of the gastric body, continuing proximally into the esophagus (Figure 3) and subsequently along distal to the pylorus (Figure 4). Part of the gastric fundus adhered to the diaphragmatic crus is totally loose in its posterior portion, freeing up all the adhesions to complete dissection of the diaphragmatic crus with ligation of the posterior gastric artery (Figure 5). With the entire dissected stomach starts clipping about 2 cm from the pylorus with green load stapler 60 mm using Echelon (Echelon Flex Endopath - Ethicon - Johnson & Johnson Corporation - USA) and without introduction of the gastric tube for this first clipping (Figure 6). The usual sequence is to follow with a golden cargo and complete the staple line with blue charges, all of 60 mm. From the second shot, all subsequent steps are done with the calibration done by gastric probe number Fr 32 inside the gastric tube, guiding the positioning direction parallel to the stapler (Figure 7). In the last shot attention to maintain approximately 0.5 to 0.8 cm stomach near the esophagogastric angle to avoid inadvertent clipping of the abdominal esophagus (Figure 8). By conducting the second and third shots, it should be observed carefully the position of the angular notch, thereby avoiding narrowing or rotation of the gastric tube at this point. Before each shot, it must be evaluated properly position the stapler in reference to the anterior and posterior stomach wall in order to construct fully symmetrical gastric tube.

**FIGURE 9:**
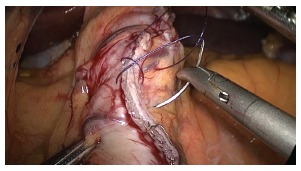
- Continuous oversuture being held in stapling line with absorbable suture in a single layer

Upon completion of the stapling and gastric tube production, is carried out continuous suture, transmural and transfixing with absorbable Caprofil(r) 3-0 (Ethicon - Johnson & Johnson Corporation - USA), who started both by the transition, as near the pylorus, with the completion of the suture in the middle portion of gastric tube body (Figures 9 and10). After leak testing of the staple line with methylene blue solution, the stomach is removed by incision of the T3 after digital dilation. This opening is sutured with absorbable Vicryl(r) 0 (Ethicon - Johnson & Johnson Corporation - USA). The abdominal cavity drainage is not performed. After review of hemostasis, surgical gauze and needles counting, the trocars are removed with direct visualization to evaluate the presence of bleeding in the holes of the portals. The skin is sutured using intradermal separate sutures of Monocryl 4-0 (Ethicon - Johnson & Johnson Corporation - USA) and the dressing is made by applying an adhesive solution to skin (Dermabond(r) - Ethicon - Johnson & Johnson Corporation - USA) .

### Postoperative

Patients are kept until they are hospitalized with adequate tolerance of oral fluids, no pain, no nausea and normal walking condition. Receive proton pump inhibitor for 90 days and prophylaxis of thromboembolic events with enoxaparin for at least 10 days beyond the hospital. Vitamin-mineral and protein supplementation is recommended for one year. Revaluations with the surgeon and the entire multidisciplinary team are held in the three months till complete two years. After this, an annual consultation is recommended.

## RESULTS

During 12 months were enrolled 120 patients undergoing SG according to the technique described herein. The results are published in another article ABCD (ABCD 2015;28(Supl.1):61-64) in this same volume/number.

## DISCUSSION

Several reasons, besides the good result of weight loss, have contributed to the worldwide acceptance of SG. It is considered operation technically easier and simpler; does not need anastomosis; none nutritional problems; and does not generate vitamin-mineral need for replacement for a long time, since there is no intestinal bypass. Although the SG was firstly proposed as a procedure with significant limits - reserved for severe cases with the intention to reduce the surgical risk - quickly these limits have been expanded, especially in patients where the realization of the Roux-en-Y gastric bypass was controversial, such as in patients with inflammatory bowel disease, previous abdominal operations and before or after organ transplantation such as liver and kidney^9^^,^ 12 ^,^ 13.

Perhaps the only consensual aspect is that the SG should preferably be done by laparoscopy because the dissection of the great gastric curvature near the spleen is greatly facilitated by direct vision that only laparoscopy can provide, avoiding iatrogenic splenic injury. Moreover, the correct stapler position near the gastroesophageal transition also can be performed by laparoscopic vision. Laparoscopic release of the greater curvature of the gastric fundus is faster and more secure with the use of ultrasonic energy or bipolar electrocautery. In this study, the preference is for the use of ultrasound (Ultracision Harmonic Ace Plus- Ethicon - Johnson & Johnson Corporation - USA) which by means of mechanical vibration energy enables the sealing of tissue by protein denaturation resulting in rapid and reliable hemostasis^5^^,^ 6 ^,^ 11.

 Among the technical controversies about SG is the diameter of the remaining stomach, especially in the gastric body region. In practice, surgeons use of a "template" intragastric for guiding the staple line, generally probe Nelaton(r) type "Fouchet". There is no consensus whether this probe should serve only as "anatomical" parameter for guidance of the staple line or whether it should be used as a calibration mold (diameter) of gastric tube. And, in case of serving as calibration template, there is no consensus about what would be the ideal size for it.

In this aspect, several evaluations have analyzed the outcome of surgery with different gastric tube calibration standards, from 28 to more than 50 Fr. Calibrations over 40 Fr have been associated with poor performance or loss of important regained weight. Apparently, adjust the calibration to less than 36 Fr does not lead to better weight loss results, but increases significantly the risk of complications, especially the leackage transition and clinically symptomatic stenoses. Also over time, oesophagogastric fistula at the angle seemed much more related to the transition fragility of local problems than in relationship with clipping too close to the angular notch. What appears to be consensus among surgeons is the need to use this intragastric "mold" to guide the staple line, as well as offering greater technical security; the absence of this parameter can result in inappropriately broad remaining stomachs and compromise weight loss^14^.

**FIGURE 10:**
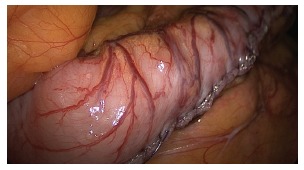
- Oversuture completed on the stapling line and final appearance of the gastric tube

An example of the technical details to be observed to achieve better results with low complication rate is the care of the staple firing in the notch region because, although it was opted for calibration and adjusted to a 32 Fr catheter, it should be prevented narrowing too much this region, thus avoiding difficulties of stomach body emptying and consequent increase in the pressure of the gastric tube, and an increased risk of leaks near the esophagogastric junction.

The use of some sort of reinforcement of the staple line is another controversial point in the technical systematization of SG. It has been quite common to use synthetic, bioabsorbable and specifically designed for application on clipping lines in laparoscopic surgery for the purpose of reducing the occurrence of bleeding and fistulas (Gore Seamguard(r); WL Gore & Associates, Inc., Flagstaff, AZ). However, the results published in the biomedical literature is conflicting because some studies have not shown advantages of oversuture with surgical thread or stapling technique without any reinforcement^1^^,^ 7 ^,^ 8.

## CONCLUSION

The surgical technique proposed here presented itself viable facilitating the surgeon's work on difficult points of the sleeve gastrectomy.
